# *park^+/+^* and *park^−/−^ Drosophila* have sexually dimorphic brain redox chemistry

**DOI:** 10.1242/dmm.052250

**Published:** 2025-08-19

**Authors:** Amber N. Juba, Bobbi Stwalley, Tigran Margaryan, Riley Hamel, Amanda N. Foley, T. Bucky Jones, Artak Tovmasyan, Lori M. Buhlman

**Affiliations:** ^1^ Biomedical Sciences Program, Midwestern University, Glendale, AZ 85308, USA; ^2^ Department of Anatomy, Arizona College of Osteopathic Medicine, Midwestern University, Glendale, AZ 85308, USA; ^3^ Department of Translational Neuroscience, Ivy Brain Tumor Center at Barrow Neurological Institute, Phoenix, AZ 85013, USA

**Keywords:** Sex, Sexual dimorphism, Parkin, Oxidative stress, Antioxidants, Mitochondria, Hydrogen peroxide, Glutathione, Dopaminergic neuron, *Drosophila*, Redox, Parkinson's disease

## Abstract

Sexual dimorphism in Parkinson's disease (PD) pathophysiology is poorly understood. Elucidating consequences of disease-causing mutations on brain redox chemistry may reveal therapeutic targets for all people with PD. We report that male *Drosophila* had increased hydrogen peroxide and glutathione (G-SH) redox disequilibrium in vulnerable dopaminergic neuron mitochondria. Levels of cysteine and oxidized cystine were decreased, with cysteine/cystine ratios (indicating less oxidative stress) and G-SH levels being elevated in parkin-null (*park^−/−^*) *Drosophila* brains, and more so in males. We report effects of parkin loss and sex on the levels of low-molecular-weight thiols involved in G-SH synthesis, providing clues as to mechanisms implicated in altered levels of brain G-SH, cysteine and cystine. Protein nitration was decreased in the brain of *park^−/−^* flies, especially in females, suggesting that decreased nitric oxide levels compensate for loss of parkin or lack of protective nitric oxide synthase activity. Our results imply that *park^−/−^* flies have elevated levels of G-SH that meet antioxidant demand in the absence of parkin in the whole brain, but not in vulnerable neurons. Identification of sexually dimorphic PD risk factors could inform symptom management and highlight sex-specific therapeutic strategies.

## Teaser

**Summary:** Sexual dimorphism in the brain redox environment may contribute to sexually dimorphic *park^−/−^ Drosophila* phenotypes. Degenerating and non-degenerating neurons have different redox environments.

## INTRODUCTION

Implications of biological sexual dimorphism throughout the lifespan and in pathophysiology are rapidly being uncovered. Regarding Parkinson's disease (PD), women are less likely to be affected, but female individuals have faster progression and higher mortality rates (Baldereschi et al., 2000; Solla et al., 2012; Dahodwala et al., 2018; Larsson et al., 2018). Sexual dimorphisms have been observed in motor and non-motor PD symptoms, and responses to symptom management also vary by sex (reviewed by Cerri et al., 2019). Mechanisms underlying dimorphisms are less clear. In addition to differences in circulating steroid hormone levels, sexual dimorphisms in redox activity may be involved, and the interplay between steroid hormone activity and redox factors is intriguing. Females and males have differential gene expression in the substantia nigra, including different ratios of dopamine 1 and 2 receptor subtypes (Cantuti-Castelvetri et al., 2007; Simunovic et al., 2010; Cullity et al., 2019), and several studies report less dopaminergic vulnerability in female rodents (Bispo et al., 2019; Marin and Diaz, 2018). Higher levels of reduced glutathione (G-SH) have been detected in brains of healthy young males, the more vulnerable sex in regard to PD etiology, and G-SH levels decline with age only in male mice (Mandal et al., 2012; Wang et al., 2003). Evidence of sex-based redox differences has largely come from non-human mammals. Male rats and sheep have elevated activity of liver glutathione reductase, which converts oxidized glutathione dimers (GS-SG) to G-SH, suggesting elevated oxidative stress (Al-Gubory and Garrel, 2016; Rikans et al., 1991). Sex-based differences in levels and activity of a variety of redox players have been detected; however, changes appear to be species and tissue -dependent, and they vary in direction of change (reviewed by Wang et al., 2020). Elucidating sexually dimorphic redox states in humans and animal disease models will improve our understanding of events that lead to onset and progression of diseases that present differently in women and men. Biological sex may, eventually, serve as a pivotal factor informing disease management.

Oxidative stress is primarily implicated in idiopathic and inherited forms of PD pathophysiology (Dias et al., 2013). Selective degeneration of dopaminergic neurons in the substantia nigra subdivision *pars compacta* causes hallmark PD motor symptoms. Interestingly, the rest of the brain is generally spared from neurodegeneration. In the *Drosophila* brain, dopaminergic neuron clusters support arousal and motor activity (Lima and Miesenböck, 2005), and the protocerebral posterior lateral 1 (PPL1) region is functionally homologous to substantia nigra in that it promotes motivated behavior (Strausfeld and Hirth, 2013; Krashes et al., 2009; Claridge-Chang et al., 2009). Parkin-null (*park^−/−^*) *Drosophila* are a powerful model of PD because they have a decreased lifespan, a severe motor phenotype and selective degeneration of PPL1 dopaminergic neurons (Whitworth et al., 2005; Houlihan et al., 2022; Cackovic et al., 2018). Oxidative stress is heavily implicated in substantia nigra and PPL1 neurodegeneration in the absence of parkin. To explore mechanisms that trigger PPL1 oxidative stress in this model, we have previously used highly selective redox-sensitive fluorescent proteins to report increased protein oxidation, hydrogen peroxide levels and G-SH redox disequilibrium in mitochondria of *park^−/−^ Drosophila* PPL1 neurons (Houlihan et al., 2022; Cackovic et al., 2018). Using similar strategies, we now report that these indices of oxidative stress were elevated to a greater extent in male compared to female flies. Using a liquid chromatography, tandem mass spectrometry (LC-MS/MS) protocol in *park^−/−^ Drosophila* brains, we also observed changes in low-molecular-weight thiols involved in glutathione synthesis and decreases in oxidative stress markers that were more pronounced in males. Interestingly, we detected decreased nitration [a marker for nitric oxide (NO) and peroxynitrite signaling] of brain proteins – particularly in females – suggesting that *park^−/−^* flies lack protective NO signaling and/or that levels of reactive nitrogen species are buffered by an antioxidant response triggered by the absence of parkin at whole-brain level.

## RESULTS

### Levels of hydrogen peroxide and the glutathione redox equilibrium are elevated in *park*^*+/+*^ and *park*^*−/−*^ male, and in *park^−/−^* female PPL1 mitochondria

**Fig. 1.**
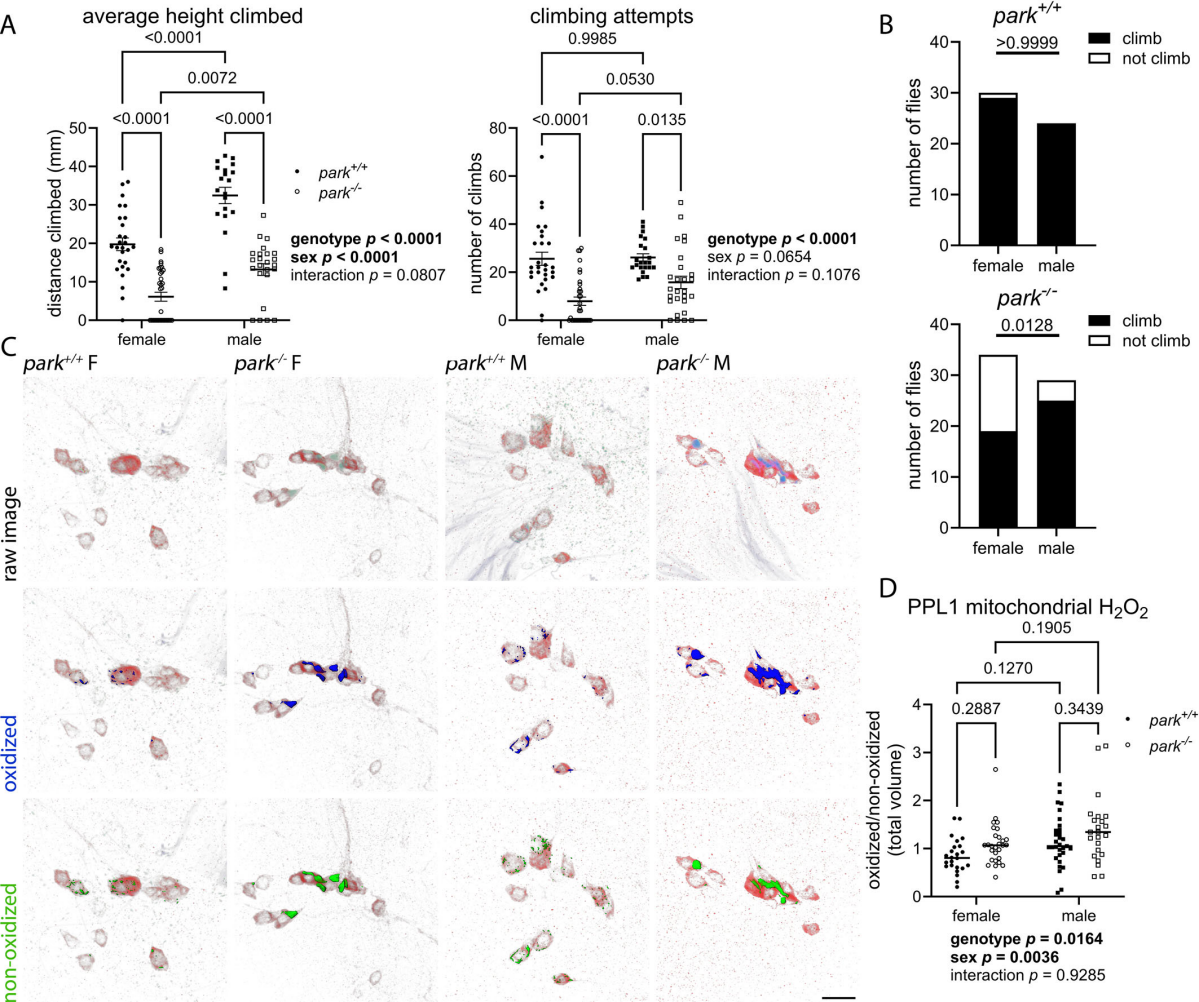
**Decreases in climbing capability and motivation in *park^−/−^ Drosophila* are accompanied by elevated levels of PPL1 mitochondrial hydrogen peroxide.** (A) Average height climbed and climbing attempts were recorded for individual flies over 20 min. Each data point represents data from one park^+/+^ or park^−/−^ fly (*n* for height climbed: *park^+/+^* females=38; *park^−/−^* females=35; *park^+/+^* male=32; *park^−/−^* male=41; *n* for climbing attempts: *park^+/+^* females=37; *park^−/−^* females=32; *park^+/+^* male=33; *park^−/−^* male=38). Data were collected from 20 cohorts. (B) Contingency graphs demonstrate that fewer female *park^−/−^* flies attempt to climb (*n*=30 for *park^+/+^* females, *n*=24 for *park^+/+^* males, *n*=34 for *park^−/−^* females, *n*=29 for *park^−/−^* males). Data were collected from eleven cohorts. (C) Representative images of PPL1 regions from female (F) or male (M) flies expressing mito-roGFP2-Orp1. Brains were dissected on days 4-6 post eclosion, and ratios of total volumes of oxidized to non-oxidized fluorophore emissions were calculated for one PPL1 region per brain. Top row: Raw images showing PPL1 regions stained for tyrosine hydroxylase (red) in flies expressing TH-driven mito-roGFP2-Orp1. Middle and bottom rows: Selected volume isosurfaces of oxidized (blue, middle row) and non-oxidized (green, bottom row) mito-roGFP2-Orp1 above threshold. Scale bar: 10 µm. (D) Each data point represents the ratio of the total oxidized/non-oxidized roGFP2 volume from one PPL1 region (*n*=25 for *park^+/+^* females, *n*=28 for *park^−/−^* females, *n*=31 for *park^+/+^* males, *n*=23 for *park^−/−^* males). Data represent five different dissection/staining experiments. Two-way ANOVA followed by Tukey's multiple comparisons tests were used for A and D. Fisher's exact tests were performed to determine effect of genotype and sex (B). Error bars represent standard error of the mean; Fisher's exact test and post-hoc *P* values are indicated above graphs. ANOVA *P* values are indicated to the right of graphs in A and below graphs in D. In panels A, B and D, circles indicate female flies; squares indicate male flies; white circles/squares indicate *park*^−/−^ flies; black circles/squares indicate *park*^+/+^ flies.

Motor deficiencies in *park^−/−^ Drosophila* appear within days of eclosion and have been well characterized (Mannett et al., 2022; Cackovic et al., 2018; Houlihan et al., 2022; Greene et al., 2003; O'Hanlon et al., 2022). PPL1 promotes motivated behavior, and we previously reported that *park^−/−^* PPL1 mitochondria have increased protein oxidation, elevated hydrogen peroxide levels and glutathione disequilibria, each of which may contribute to climbing deficits (Cackovic et al., 2018; Houlihan et al., 2022; Strausfeld and Hirth, 2013). *park^−/−^* flies have deficits in both ability and motivation to climb, as indicated by average-height climbed and climbing-attempt studies, respectively (Houlihan et al., 2022; Mannett et al., 2022). To explore whether these effects are sexually dimorphic, we repeated our studies and found that, compared with *park^+/+^* flies, *park^−/−^* flies of either sex have decreased climbing ability and motivation (Fig. 1A, two-way ANOVA, *P*<0.0001 for height climbed and climbing attempts), and that the effect on climbing capability is driven by the female sex (Fig. 1A, two-way ANOVA, *P*<0.0001; Fig. 1B, *P* value for Fisher's exact test=0.0128).

**Fig. 2.**
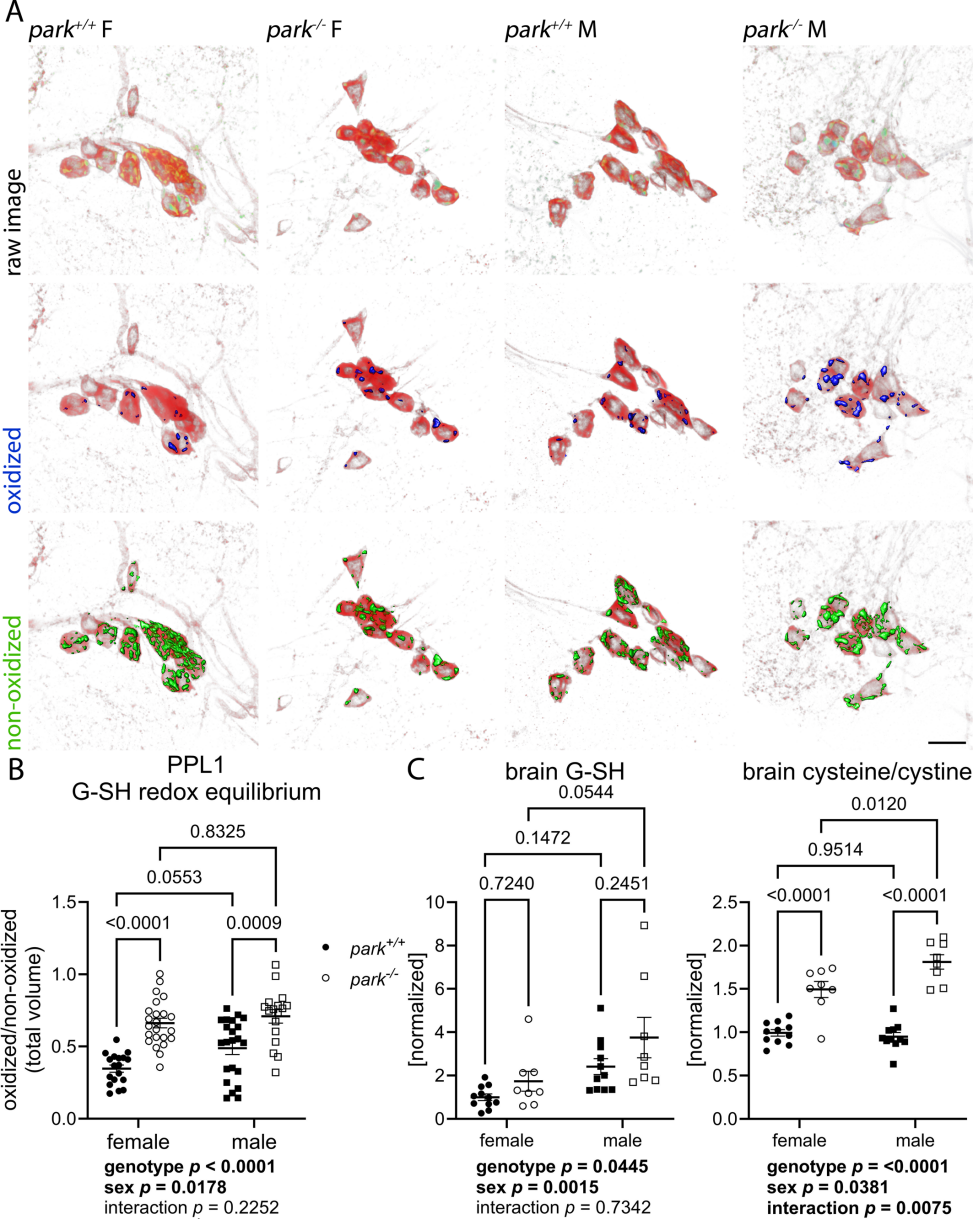
**Vulnerable PPL1 neurons in *park*^*+/+*^ and *park*^*−/−*^ males, and *park^−/−^* females have glutathione disequilibrium, while whole-brain oxidation indicators are decreased.** (A) Representative images of PPL1 regions from flies expressing mito-roGFP2-Grx1. Brains were dissected on days 4-6 post eclosion and ratios of total volumes of oxidized to non-oxidized fluorophore emissions were calculated for one PPL1 region per brain. Top row: raw images showing PPL1 regions stained for tyrosine hydroxylase (red) in flies expressing TH-driven mito-roGFP2-Grx1. Middle and bottom rows: selected volume isosurfaces of oxidized (blue, middle row) and non-oxidized (green, bottom row) mito-roGFP2-Grx1 above threshold. Scale bar: 10 µm. (B) Each data point represents the ratio of the total oxidized/non-oxidized total roGFP2 volume from one PPL1 region (*n*=18 for control (*park^+/+^*) females, *n*=23 for parkin-null (*park^−/−^*) females, *n*=22 for *park^+/+^* males, *n*=17 for *park^−/−^* males). Data represent eleven different dissection/staining experiments. (C) *park*^*+/+*^ and *park*^*−/−*^ males, and *park^−/−^* females have elevated antioxidant markers at the whole-brain level. *park^+/+^* and *park^−/−^* fly heads were collected and frozen on days 4-6 post eclosion. LC-MS/MS was performed to detect reduced levels of glutathione (G-SH), cysteine and cystine. The increased ratio of cysteine to cystine in *park*^*−/−*^ males and females indicates a more-reduced redox environment. Each data point represents one tube of homogenate from a pool of about 20 brains (*n*=11 for *park^+/+^* females, *n*=8 for *park^−/−^* females, *n*=11 for *park^+/+^* males, *n*=8 for *park^−/−^* males). Samples for each genotype were continuously harvested for 1 year. LC-MS/MS was performed on three groups of samples on three different dates. (B,C) Two-way ANOVA followed by Tukey's multiple comparisons test were performed to determine effect of genotype and sex. Error bars represent standard error of the mean. ANOVA and post-hoc *P* values are indicated below and above graphs, respectively. In panels B and C, circles indicate female flies; squares indicate male flies; white circles/squares indicate *park*^−/−^ flies; black circles/squares indicate *park*^+/+^ flies.

Our results support previous work demonstrating that male *Drosophila* generally have superior climbing capabilities (Aggarwal et al., 2019; Watanabe and Riddle, 2021). We found that *park^−/−^* males also have improved climbing capability compared to *park^−/−^* females (Fig. 1A left graph, Tukey's multiple comparisons test, *P*=0.0072), but no indication of sex affecting climbing motivation (Fig. 1A right graph, two-way ANOVA, *P*=0.0654). The effects of genotype on PPL1-driven climbing motivation are reflected by disrupted PPL1 mitochondrial redox status, i.e. elevated hydrogen peroxide levels and G-SH redox equilibrium in *park^−/−^* flies (Fig. 1C,D, two-way ANOVA, *P*=0.0164 for hydrogen peroxide; Fig. 2A and B, two-way ANOVA, *P*<0.0001 for G-SH redox equilibrium). However, while males had elevated PPL1 mitochondrial stress markers, these were not associated with comparably decreased climbing motivation (Fig. 1D, two-way ANOVA, *P*=0.0036 for hydrogen peroxide; Fig. 2B, two-way ANOVA, *P*=0.0178 for G-SH redox equilibrium). Using LC-MS/MS on young, mixed-sex *park^−/−^* fly brain homogenates, we previously reported no change in the levels of G-SH, a marker of oxidative stress (Houlihan et al., 2022). When young flies were separated by sex, however, brain G-SH levels and cysteine/cystine ratios were elevated by the absence of parkin (Fig. 2C, two-way ANOVA, *P*=0.0445 for G-SH and *P*<0.0001 for cysteine/cystine), and the effect on cysteine/cystine ratios is exacerbated in *park^−/−^* males (Fig. 2C, Tukey's multiple comparison's test comparing sex in *park^−/−^* flies, *P*=0.0120; two-way ANOVA genotype-sex interaction, *P*=0.0075). Male G-SH levels were elevated (Fig. 2C, two-way ANOVA, *P*=0.0015), further indicating that the redox environment in the male brain is more reduced. Thus, loss of parkin caused oxidative stress markers to be elevated in vulnerable PPL1 dopaminergic neurons, while the redox environment in non-degenerating cells of the central brain was reduced. Our results support the possibility that parkin loss triggers an antioxidant response in the brain of young flies that is not achieved by PPL1 dopaminergic neurons, rendering them vulnerable to degeneration.

### Levels of select low-molecular-weight thiols in *park*^−/−^ and *park*^+/+^ *Drosophila* brains are dysregulated in a sex-dependent manner

**Fig. 3.**
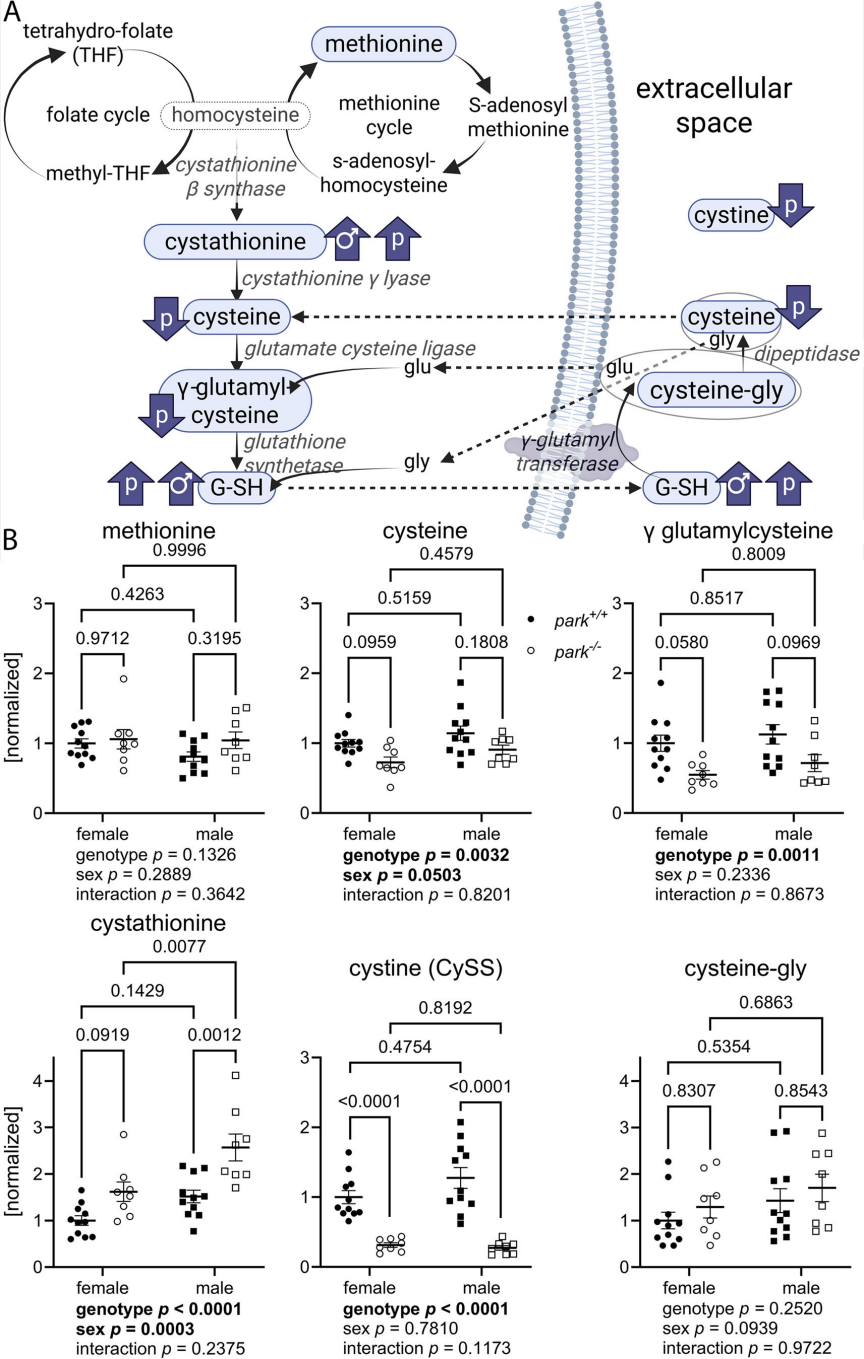
**Transsulfuration pathway components are disrupted in brains of *park*^*+/+*^ and *park*^*-/-*^ male, and *park^−/−^* females.** (A) Transsulfuration pathway of glutathione (G-SH) synthesis. Measured low-molecular-weight (LMW) thiols are within blue ovals. Blue arrows indicate direction of change in female and male *park^−/−^* flies (*P*) and *park*^*+/+*^ males (♂). Enzymes are italicized. Essential amino acid methionine and tetrahydro-folate from dietary folate feed into the transsulfuration pathway of G-SH synthesis. G-SH is transported out of the cell where it is broken down into cysteinylglycine (cysteine-gly) dimers, which are further broken down to cysteine and glycine (gly) by dipeptidase. Amino acids are transported into the cell where they can enter the transsulfuration pathway as indicated by the arrows. Solid arrows indicate enzymatic reactions; dashed arrows indicate movement across the plasma membrane; gly, glycine; glu, glutamate. (B) *park*^*+/+*^ and *park*^*−/−*^ males, and *park^−/−^* females have elevated cystathionine, and female and male *park^−/−^* flies have decreased cysteine, cystine (CySS, oxidized cysteine), and γ glutamylcysteine. *park^+/+^* and *park^−/−^* fly heads were harvested and frozen on days 4-6 post eclosion. Each data point represents one tube of homogenate from a pool of approximately twenty brains (*n*=11 for *park^+/+^* females, *n*=8 for *park^−/−^* females, *n*=11 for *park^+/+^* males, *n*=8 for *park^−/−^* males). Samples for each genotype were continuously harvested for one year; LC-MS/MS was performed on three groups of samples on three different dates. Two-way ANOVA followed by Tukey's multiple comparison's tests were performed to determine effect of genotype and sex. Error bars represent standard error of the mean; ANOVA and post-hoc *P* values are indicated below and above graphs, respectively. In panel B, circles indicate female flies; squares indicate male flies; white circles/squares indicate *park*^−/−^ flies; black circles/squares indicate *park*^+/+^ flies. Created in BioRender. Buhlman, L. (2025) https://BioRender.com/qti62ct.

To address whether genotype and sex differences in brain redox status could be influenced by changes in the transsulfuration pathway for glutathione synthesis, we performed LC-MS/MS on pooled brain homogenates and measured levels of relevant low-molecular-weight thiols (Fig. 3A). Methionine and cysteinyl-glycine (cysteine-gly) enter the transsulfuration pathway at different points (Fig. 3A), and their levels are unaffected by genotype or sex (Fig. 3B, methionine two-way ANOVA, *P*=0.1326 for genotype, *P*=0.2889 for sex; cysteine-gly two-way ANOVA, *P*=0.2520 for genotype, 0.0939 for sex). However, several notable changes were observed in other low-molecular-weight thiol metabolites. Cystathionine, a metabolite downstream of homocysteine, was upregulated in female and male *park^−/−^* flies, and in *park^−/−^* males compared to *park^−/−^* females (Fig. 3B, two-way ANOVA, *P*<0.0001 for genotype, *P*=0.0003 for sex). The increase in cystathionine is particularly intriguing, as levels of downstream metabolites, including cysteine, cystine and γ-glutamylcysteine, were decreased in *park^−/−^* brains, with no influence of sex (Fig. 3B, two-way ANOVA, genotype, *P*=0.0032, *P*<0.0001 and *P*=0.0011, respectively). Decreases in cystine and γ-glutamylcysteine could directly result from reduced cysteine availability, with elevated cystathionine possibly representing a compensatory effort to sustain cysteine and downstream metabolites. The decrease in these metabolites might also indicate impaired enzymatic conversion from cystathionine to cysteine and its related metabolites.

Despite diminished cysteine and related metabolite levels, we detected increased G-SH levels (Fig. 2C), suggesting a compensatory mechanism to offset reduced cysteine levels through increased G-SH synthesis for oxidative stress mitigation. The simultaneous rise in cystathionine and G-SH levels, alongside diminished cysteine concentrations, implies that metabolic dynamics may be shifting towards glutathione synthesis, potentially at the cost of free cysteine availability – possibly due to heightened utilization of cysteine for protein synthesis or in response to elevated oxidative stress. These findings indicate a complex regulatory framework within the transsulfuration pathway. Furthermore, the balance between cysteine used for protein synthesis and its role in neutralizing reactive species, thereby influencing glutathione synthesis, may explain fluctuations of redox homeostasis in *park^+/+^* versus *park^−/−^* fly brains. Further investigations are necessary to clarify these interactions and to discern whether they signify adaptive responses to oxidative stress or shifts in metabolic flux.

### Nitration of brain proteins, but not nitric oxide synthase, is decreased by parkin loss of function – particularly in females

**Fig. 4.**
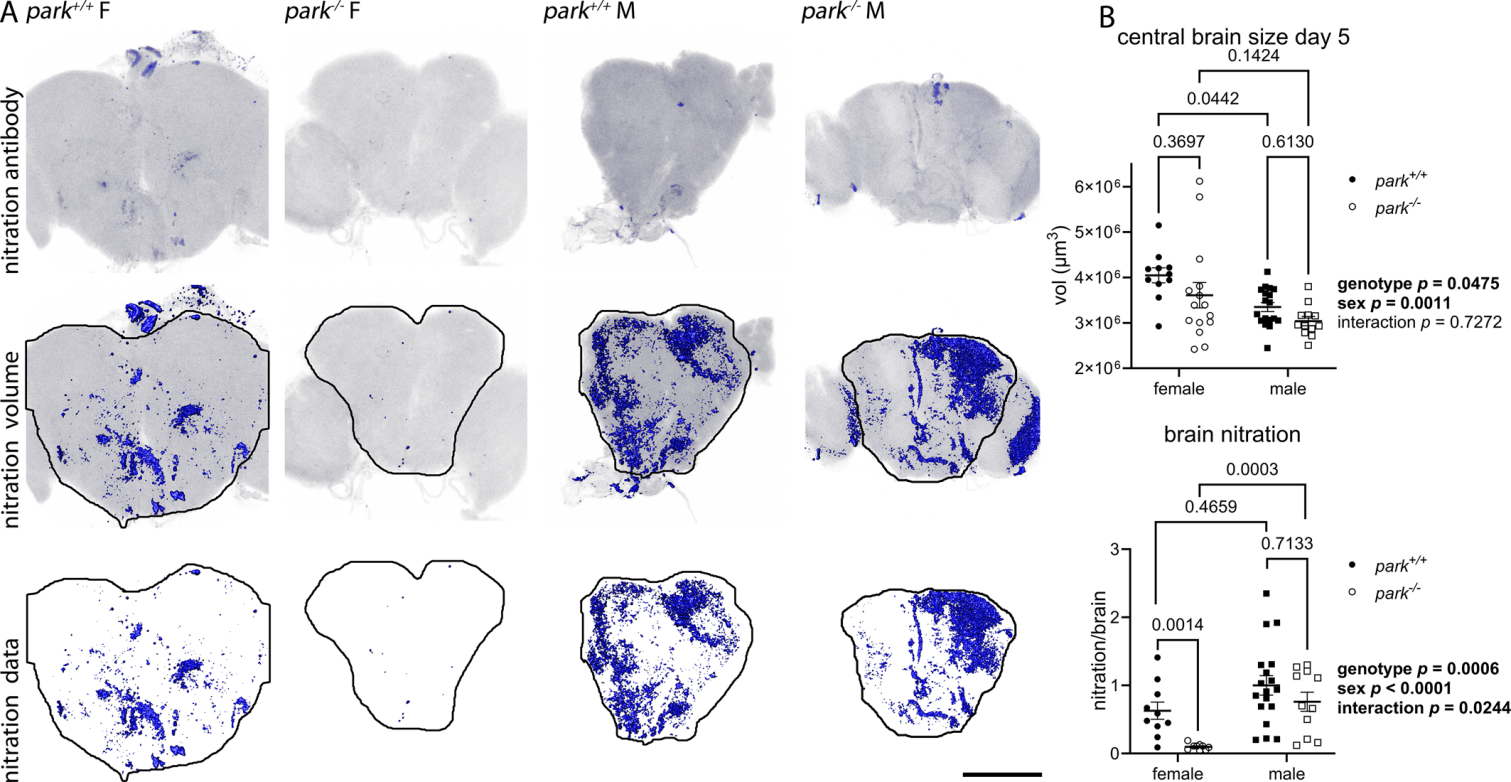
**Central brain nitration levels are elevated in *park*^*+/+*^ and *park*^*−/−*^ males, and decreased in *park^−/−^* females.** Brains were dissected and total volume of central brain anti-tyrosine nitration antibody emission was calculated for flies aged 4 to 6 post eclosion. (A) Representative 2D-images of the central brain (encircled) obtained from female (F) or male (M) *park^+/+^* and *park^−/−^* flies. Blue isosurfaces indicate anti-nitrotyrosine antibody staining above background. Top row: raw images. Middle row: whole-brain antibody emission above background. Bottom row: central brain antibody emission above background. Scale bar: 200 μm. (B) Top graph: Central brain volume is decreased in *park*^*+/+*^ and *park*^*−/−*^ males, and *park^−/−^* females. Bottom graph: Brain nitration is decreased by the *park* mutation and by the female sex. Each point represents data for one brain (for brain size data, *n*=11 for *park^+/+^* females, *n*=15 for *park^−/−^* females, *n*=18 for *park^+/+^* males, *n*=12 for *park^−/−^* males; for nitration data, *n*=10 for *park^+/+^* females, *n*=9 for *park^−/−^* females, *n*=18 for *park^+/+^* males, *n*=11 for *park^−/−^* males). Data represent five different dissection/staining experiments. Circles indicate female flies; squares indicate male flies; white circles/squares indicate *park*^−/−^ flies; black circles/squares indicate *park*^+/+^ flies. Two-way ANOVA followed by Tukey's multiple comparisons tests were performed to determine the effect of genotype and sex. Error bars represent standard error of the mean; ANOVA and post-hoc *P* values are indicated to the right and above graphs, respectively.

Brain nitration is a post-translational modification (PTM) capable of altering protein structure and function. It is increasingly recognized as a prevalent PTM in PD, with implications for its pathogenesis (reviewed by Stykel and Ryan, 2022). Nitration occurs when peroxynitrite reacts with tyrosine amino acid residues as a feature of innate immune and homeostatic NO signaling (Giannopoulou et al., 2002; Reinehr et al., 2004; Di Stasi et al., 1999). Tyrosine residue nitration reflects the level of peroxynitrite produced as a result of NO, generated by NO synthase (NOS) interacting with superoxide anions. Several proteins relevant to PD and/or glutathione metabolism can be nitrated; these include tyrosine hydroxylase (TH), α-synuclein, superoxide dismutase 1 (Sod1, also known as CuZn), manganese superoxide dismutase, glutathione reductase, glutaredoxin, glutathione-S-transferase and cytochrome c oxidase (reviewed by Szabó et al., 2007; and Danielson and Andersen, 2008). Changes in glutathione metabolism can influence the level of glutathionylation, a process through which G-SH binds to cysteine residues under oxidative stress conditions. Glutathionylation of NOS can alter the level of its phosphorylation, i.e. its activity, uncoupling it from NO production (reviewed by Kavya et al., 2006). Mammals express three isoforms of the *nitric oxide synthase* gene (*NOS*), inducible NOS (iNOS), neuronal NOS (nNOS) and endothelial NOS (eNOS). The nNOS and eNOS isoforms are constitutively expressed and dependent on Ca^2+^ levels, while the iNOS isoform is induced in response to inflammatory stimuli and regulated independently of Ca^2+^. In contrast, *Drosophila* have one *Nos* gene, and the *Drosophila* NOS (dNOS) enzyme can facilitate mammalian iNOS- and nNOS-like signaling (Stasiv et al., 2001; Ajjuri and O'Donnell, 2013). We explored whether NO and peroxynitrite levels are sexually dimorphic and/or elevated in *park^−/−^* brains as in an inflammatory response (Ajjuri and O'Donnell, 2013). We incubated dissected brains by using 1% nitrotyrosine polyclonal antibody purified from rabbit serum (#21285, ThermoFisher Scientific, Waltham, PA, USA) that detects nitrated tyrosine amino acid residues and found it to be decreased in *park^−/−^* flies (Fig. 4A,B, two-way ANOVA, *P*=0.0006), and almost absent in *park^−/−^* females (Fig. 4A,B). There was an effect of sex and an interaction (Fig. 4B, two-way ANOVA, *P*<0.0001 and *P*=0.0244, respectively) indicating that nitration in *park^−/−^* males is elevated compared with *park^−/−^* females, an effect that was not observed in *park^+/+^* flies (Fig. 4A,B, Tukey's Multiple Comparisons test, *P*=0.4659 for *park^+/+^* and *P*=0.0003 for *park^−/−^*). Male *Drosophila* are smaller than females (Matthews et al., 2005); accordingly, we observed decreased central brain volume in males and smaller brain volume in *park^−/−^* flies (Fig. 4B, two-way ANOVA, *P*=0.0011 for sex, *P*=0.0475 for genotype). Therefore, we controlled for brain volume in our antibody analyses of protein nitration.

**Fig. 5.**
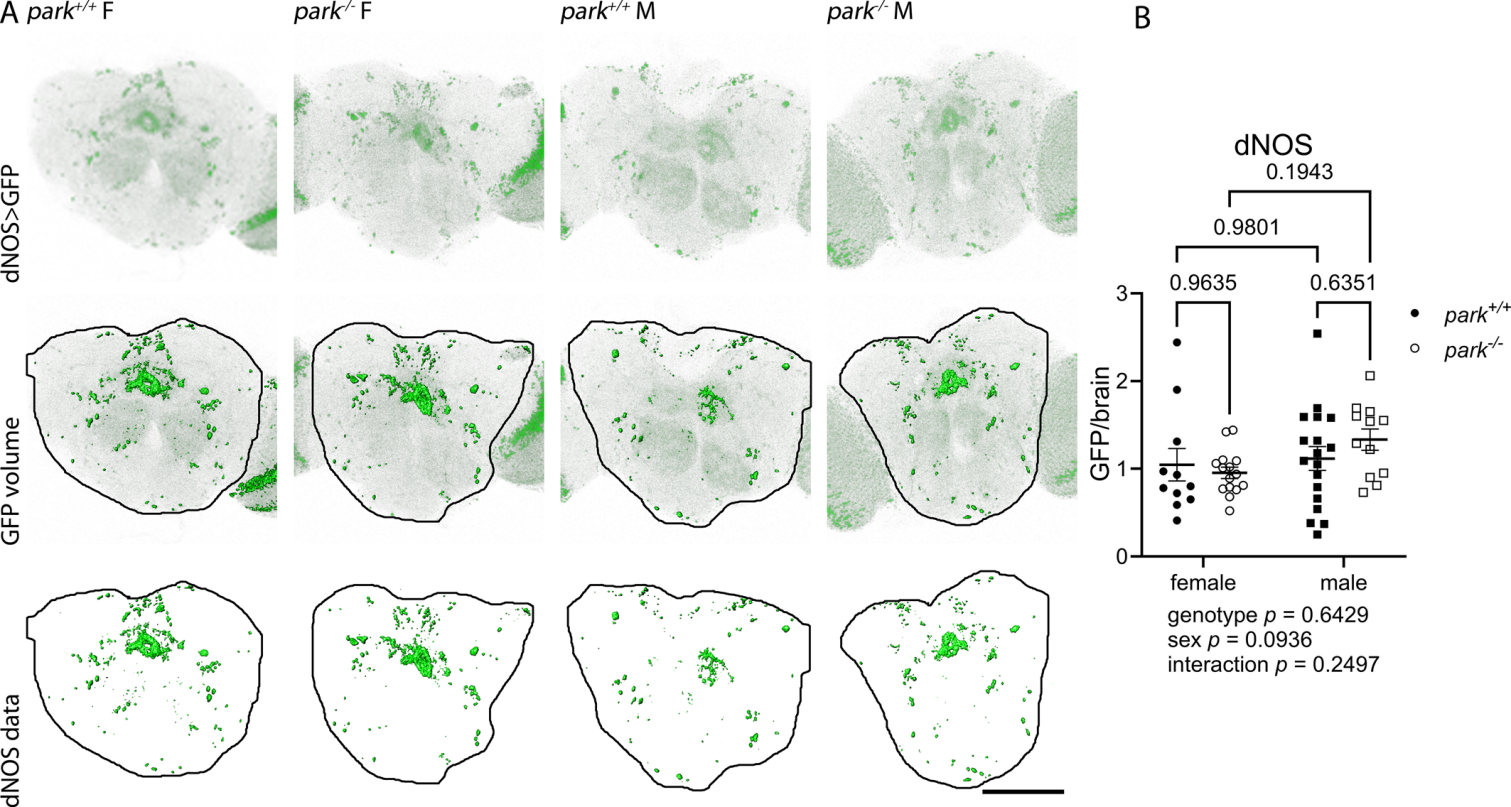
**DNOS levels are unaffected by sex or parkin loss of function.** (A) Representative 2D-images of the central brain (encircled) showing the dNOS-driven EGFP volume. Brains were dissected from day 4 to 6 post-eclosion *park^+/+^* and *park^−/−^* flies expressing dNOS-driven EGFP, and the total volume of central brain EGFP emission was calculated. Green ‘isosurfaces’ indicate EGFP expression above background. Top row: raw images from flies expressing NOS-driven GFP. Middle row: EGFP expression above background. Bottom row: Total volume of central brain EGFP expression. Scale bar: 200 μm. (B) Plotted is the total fluorescence volume per central brain. Each point represents data from one fly (*n*=11 for *park^+/+^* females, *n*=15 for *park^−/−^* females, *n*=18 for *park^+/+^* males, *n*=12 for *park^−/−^* males). Circles indicate female flies; squares indicate male flies; white circles/squares indicate *park*^−/−^ flies; black circles/squares indicate *park*^+/+^ flies. Data represent five different dissection/staining experiments. Two-way ANOVA following by Tukey's multiple comparisons tests were performed to determine the effect of genotype and sex. Error bars represent standard error of the mean; ANOVA and post-hoc *P* values are indicated below and above graphs, respectively.

To address whether changes in protein nitration could be a function of altered iNOS-like peroxynitrite production, we measured the EGFP volume within the central brain of flies that express dNOS-driven EGFP. Transcription of 2×EGFP occurs in the cells of these flies when endogenous *Nos* transcription is initiated. Thus, measurements of EGFP emission are a proxy for relative *Nos* (*dNOS*) transcription. Our data imply that, unlike iNOS, dNOS activity is not transcriptionally regulated (Fig. 5, two-way ANOVA, *P*=0.0936 for sex, *P*=0.6429 for genotype). However, if decreased brain protein nitration reflects a protective response to the absence of parkin, our data may support iNOS-like activity where changes in dNOS transcription were not detected using our approach. By contrast, our data may reflect a more nNOS-like function of dNOS. Mammalian brain nNOS is constitutively expressed and its activity is Ca^2+^ dependent. dNOS has the highest sequence homology with nNOS, it can be activated by Ca^2+^, and it is involved in synapse remodeling during development and in memory formation in adult flies (Rabinovich et al., 2016; Kuntz et al., 2017; Stasiv et al., 2001).

## DISCUSSION

### Mammalian redox and steroid hormone signaling interplay

We observed sexually dimorphic alterations in redox factors within whole brains and PPL1 dopaminergic mitochondria of *park^+/+^* and *park^−/−^ Drosophila*, a powerful model of PD and other human disorders. Intriguingly, the nature of redox alteration patterns is largely consistent for *park*^*+/+*^ and *park*^*−/−*^ males, and *park^−/−^* females, suggesting sexually dimorphic redox environments that may predispose males to PD can be explored in *Drosophila*. Oxidative stress is heavily implicated in PD pathophysiology, and male rodent brain mitochondria have elevated oxidative stress compared to females, regardless of age and estrous cycle status (Gaignard et al., 2015; Guevara et al., 2011). Our PPL1 results in *Drosophila* are congruent with those of post-mortem examinations in humans (Gaignard et al., 2015; Guevara et al., 2011), and with experimental model reports indicating that mitochondria in the neurons of female rodents show lower oxidative stress than those in male rodents, independent of age and estrous cycle (Gaignard et al., 2015; Guevara et al., 2011). Mammalian estrogen receptor activity promotes expression of antioxidant enzymes glutathione peroxidase and manganese superoxide dismutase (Borrás et al., 2005). Estrogen activity is associated with increased antioxidant capacity and reduced oxidative stress (reviewed by Lejri et al., 2018); interestingly, estrogen receptor beta (ERβ) levels are elevated in a subset of rat substantia nigral neurons (Kritzer and Creutz, 2008). Brain estrogen receptor levels also vary by brain region in a sexually dimorphic manner (van Veen et al., 2020; Cao and Patisaul, 2013). In mammals, estrogen promotes dopamine synthesis, release and turnover; however, dopamine production and release can also be estrogen independent (Conway et al., 2019).

### *Drosophila* redox and steroid hormone signaling interplay

To date, few studies have addressed sex differences in *Drosophila* redox systems. *Drosophila* do not synthesize estrogens, but ecdysteroids are *Drosophila* steroid hormones that modulate a variety of heterodimeric nuclear receptor subtypes (Koelle et al., 1991; Yao et al., 1992). Conversion of ecdysone to 20-hydroxyecdysone occurs at elevated rates in mature follicle cells and is required for ovulation (Knapp and Sun, 2017). While 20-hydroxyecdysone functions similarly to progesterone, *Drosophila* express one estrogen-related receptor ortholog (ERR) that is expressed in both sexes, and is involved in energy metabolism and mitochondrial biogenesis (Tennessen et al., 2011; Beebe et al., 2020; Misra et al., 2017; Gupta et al., 2022). *Drosophila* ERR activity is sexually dimorphic in that it promotes female germline stem cell maintenance and spermatogenesis; thus, it appears that its regulation upstream is sexually dimorphic (Zike et al., 2025; Huang et al., 2023). Unlike humans or mice, *park^−/−^* male *Drosophila* are sterile due to incomplete spermatid development. Reproductive phenotypes of *park^−/−^*might be unique to *Drosophila*; however, inefficient *park^−/−^* fly spermatid development is thought to result from mitochondrial dysfunction (Greene et al., 2003). Thus, the effects of parkin on mitochondrial function and cellular homeostasis, and its interplay with steroid hormone receptors might be conserved. Literature regarding sexually dimorphic regulation of *Drosophila* ecdysone receptor (EcR) in the brain is lacking; however, it is plausible that ecdysone signaling may be differentially regulated as it is in developing gonadal tissue. Expression of *EcR* is regulated by the doublesex transcription factor that regulates gonad development in all animals (Grmai et al., 2024 preprint). *EcR* expression is repressed in the testis when it initiates morphogenesis in the developing ovary; ectopic activation of ecdysone signaling in testis triggers development of follicle-like cells and ERR signaling promotes spermatid development (Grmai et al., 2024 preprint; Misra et al., 2017).

### Interplay of NO and steroid hormone signaling

NO and protein nitration promote homeostatic signaling as well as inflammatory responses in mammals and *Drosophila* (Jeong, 2024). To date, sexual dimorphism in *Drosophila* NO activity is underinvestigated*;* however, dimorphic homeostatic and stress-related NO signaling has been described in mammals. In rodents, NOS levels fluctuate with estrous cycles in an estrogen-dependent manner during synaptogenesis and are also influenced by androgen signaling (Hu et al., 2012; Dachtler et al., 2012; Singh et al., 2000). Regulation of male nNOS may depend on the glucocorticoid, corticosterone system (Zhou et al., 2011; Hu et al., 2012). Brain protein S-nitrosylation, a distinct PTM involved in NO signaling, is also sexually dimorphic in mice (Khaliulin et al., 2020). Implications of sex-based differences in NO-signaling pathways are largely undiscovered, especially in *Drosophila*, and our data suggest that their roles in synapse formation and maintenance is compromised in PD-related dopaminergic neurodegeneration. The interplay between glutathionylation and nNOS activity further complicates studies addressing the role of parkin and redox in neurodegeneration (Takata et al., 2020). In our present study, changes in nitration were not reflected by changes in dNOS expression, suggesting that dNOS activity is regulated more like nNOS that – unlike iNOS – is not transcriptionally activated. Nonetheless, it may be that nitration changes observed by us result from decreased iNOS-like activity if dNOS and iNOS -mediated inflammatory signaling are differentially modulated. In this case, decreased nitration would be congruent with observations of a more-reduced whole-brain redox environment in *park^−/−^* flies. It is also possible that our method is not sensitive enough to detect changes in dNOS expression.

### Does decreased antioxidant capacity contribute to vulnerability of the PPL1 region?

Selective PPL1 neurodegeneration in the absence of parkin allows comparative analyses of vulnerable and non-/less-vulnerable neurons. Here, we report that *park^−/−^* PPL1 mitochondrial oxidative stress markers are elevated in a sexually dimorphic manner, while oxidative stress at whole-brain level is decreased, especially in males. Perhaps our results reflect an unknown mechanism by which PPL1 is vulnerable compared to other brain regions. Like the substantia nigra, activity of the PPL1 region promotes motivated behavior, and it seems to be less important for metrics like speed (Krashes et al., 2009; Claridge-Chang et al., 2009). Our data indicate that motivation to climb (i.e. climbing attempts) is decreased in the absence of parkin and is unaffected by sex. Thus, increased levels of PPL1 oxidative stress markers are associated with decreased climbing motivation when comparing genotypes, but not when comparing sexes. Interestingly, G-SH is decreased in the substantia nigra of patients with PD but not in other brain regions, suggesting that differences in antioxidant capacity contribute to selective dopaminergic neuron vulnerability (Sian et al., 1994). LC-MS/MS experiments measuring G-SH in isolated PPL1 neurons are not feasible; however, we have previously demonstrated that the G-SH redox equilibrium within a non-degenerating dopaminergic neuron cluster (i.e. posterior protocerebral medial region 3) is similar to that in *park^+/+^* flies (Houlihan et al., 2022). While decreases in protein nitration might support a reduction in oxidative stress, decreased NO signaling might also indicate activation of axon-remodeling pathways via modulation of ecdysone-induced protein 75B (Eip75B) nuclear receptor (Rabinovich et al., 2016). Protein nitration can decrease activities of glutathione reductase and TH; thus, decreased nitration in the absence of parkin may indicate signaling towards upregulation of these enzymes (Savvides et al., 2002; Blanchard-Fillion et al., 2001). Future projects will need to explore brain redox environments for aged *park^−/−^* flies to determine whether the redox status shifts to a more-oxidative state.

## CONCLUSION

The role of oxidative stress in neurodegenerative disease has been appreciated for decades; identification of specific redox factors that trigger and/or contribute to neurodegenerative disease may play an integral part in diagnosis and treatment strategies. Further, sexual dimorphism may be one of the primary factors regarding disease etiology, presentation and progression. Mammalian brain redox environments are sexually dimorphic, as are incidence rates and symptomologies of PD. Using a powerful *Drosophila* model of PD, we have demonstrated that vulnerable dopaminergic neuron mitochondrial hydrogen peroxide and mitochondrial G-SH redox equilibrium are elevated in males and in the absence of parkin. Interestingly, the redox environment at whole-brain level was more reduced in *park*^*+/+*^ and *park*^*−/−*^ male, and in *park^−/−^* female flies. Sexually dimorphic PPL1 and whole-brain redox environments are potentially relevant, and this relevance – or the degree of relevance – should be validated in future studies. Nonetheless, our work contributes to the expanding evidence that *Drosophila* are an inexpensive and, potentially, suitable model for pre-clinical studies addressing sexual dimorphism in redox systems and disease.

## MATERIALS AND METHODS

### *Drosophila* maintenance and genotypes

*Drosophila* were raised on standard food of cornmeal and molasses at 25°C in 12/12 h light/dark cycle with constant humidity. Newly eclosed adult flies were collected within 24 h of eclosion and maintained on fresh food vials. Control (*park^+/+^*) (*w^1118^*, Bloomington Stock Center, Bloomington, IN, USA) and *park^−/−^* flies [*park^25^/park^25^*; a gift from Leo Pallanck (University of Washington, Seattle, USA)] (Greene et al., 2003) backcrossed with *park*^*+/+*^ were used for climbing and thiol/disulfide analyses. Full genotype for control is *w^1118^*; *+^w1118^*; *+^w1118^*, where ‘+^w1118^’ indicates a chromosome from the control *w^1118^* stock. The *park^−/−^* genotype is *w^1118^*; +^w1118^; *park^25^/park^25^*). For hydrogen peroxide and G-SH redox equilibrium aims, control and *park^−/−^* flies expressed redox-sensitive (ro) green fluorescent protein 2 (GFP2) fused to *C. elegans* oxidant receptor peroxidase 1 (Orp1) or human glutaredoxin 1 (Grx1) (*UAS-mito-roGFP2-Orp1* or *UAS-mito-roGFP2-Grx1*, respectively) under the control of tyrosine hydroxylase (TH) expression using the GAL4>UAS expression system (*THGAL4>UAS-mito-roGFP2-Grx1/Orp1*) (Gutscher et al., 2008, 2009; Albrecht et al., 2011; Buhlman et al., 2021). The addition of the mitochondrial localizing sequence directly transports the reporters to the mitochondrial matrix (Albrecht et al., 2011). The genotype for the control hydrogen peroxide reporter is *w^1118^*; *+^w1118^*/*UAS-mito-roGFP2-Orp1*; *+^w1118^*/*TH-GAL4*. The *park^−/−^* genotype is *w^1118^*; *+^w1118^*/*UAS-mito-roGFP2-Orp1*; *TH-GAL4*, *park^25^/park^25^*. G-SH redox equilibrium reporting genotypes are similar, except UAS-mito-roGFP2 is fused to Grx1, rather than Orp1. Control and *park^−/−^* flies expressing enhanced green fluorescent protein (EGFP) under control of the *Drosophila* nitric oxide synthase (*dNOS*) promoter were used for dNOS and protein nitration studies. The *Drosophila* line carrying NOS-GAL4 (insertion allele Scer\GAL4^Nos−MI15126-TG4.2^; Flybase ID: FBti0195496) was generated by the Gene Disruption Project as part of the Recombinase-Mediated Cassette Exchange-MiMIC Trojan-GAL4 insertion line collection (Lee et al., 2018). The gene trap cassette contains a *Trojan GAL4* exon that includes the *GAL4*-coding sequence. The cassette was inserted into a coding intron of *Nos* so that *GAL4* transcription is controlled by the regulatory sequences of the trapped *Nos* gene. Transcription of 2×*EGFP* occurs when endogenous *Nos* transcription is initiated. Thus, our GFP emission measurements are a proxy for relative *Nos* (*dNOS*) transcription. The *Trojan GAL4* exon also contains an hsp70 transcription termination signal that is predicted to disrupt *Nos* expression (i.e. flies in this study are heterozygous NOS loss-of-function mutants). Full genotypes are *w^1118^*; NOS-GAL4/*+^w1118^*; *UAS-EGFP/+^w1118^* and *w^1118^*; NOS-GAL4/*+^w1118^*; *UAS-EGFP*, *park^25^/park^25^*. RoGFP2, TH-GAL4, NOS-GAL4, and EGFP -containing stocks were purchased from the Bloomington Stock Center (mito-roGFP2-Grx1, #67664; mito-roGFP2-Orp1, #67667; TH-GAL4, #8848; NOS-GAL4, #76766 [discontinued]; UAS-2XEGFP, #6658).

### Climbing assay

Climbing assays were performed using an MB5 Multibeam Activity Monitor (TriKinetics Inc. Waltham, MA). At day 5 post eclosion individual *park^+/+^* and *park^−/−^* flies were placed into transparent 80-mm-long vertically oriented polycarbonate tubes. Seventeen infrared beams pass through each; the distance between the first and last beam is 51 mm. The MB5 records a ‘count’ each time a fly crosses an infrared beam so that the position of the fly is collected each second for 20 min. A ‘climbing attempt’ is reported when a fly initiates an ascent, and we report this as a motivated behavior. To measure climbing capability, we report ‘height climbed’, which is the distance of the continuous trajectory of a fly from one position to a higher position in the tube. Each time a fly climbs up again after moving downwards, a new ‘attempt’ and ‘height climbed’ is recorded. To calculate average height climbed, the total height climbed during the 20-min recording period was divided by number of climbing attempts. Data for two to eight flies per genotype were collected simultaneously. Sample size was calculated using preliminary data means and standard deviations assuming 0.05 for α and 0.8 for desired power. We used the robust regression and outlier removal (ROUT) method with a false discovery rate of 1% to remove outliers (GraphPad Prims 10; GraphPad Software, La Jolla, CA, USA) (Motulsky and Brown, 2006). Each data point represents activity of one fly (*n*≥24); means and standard errors of the mean are also plotted for grouped data. We performed Brown−Forsythe tests and found no differences in standard deviations (*P*=0.1558 for attempts, *P*=0.7291 for height climbed). Two-way ANOVA followed by Tukey's multiple comparisons test were performed to determine the effect of genotype and sex (GraphPad Prism 10). Fisher's exact test was performed to determine the effect of genotype and sex on whether flies made a climbing attempt during the twenty-minute recording session (GraphPad Prism 10).

### *Drosophila* brain dissection and immunofluorescence

On days 4-6 post eclosion, *park*^*+/+*^ and *park^−/−^* flies expressing mito-roGFP2-Orp1, mito-roGFP2-Grx1 or NOS-driven GFP were anesthetized with CO_2_ and brains were dissected in 1× phosphate-buffered saline (ThermoFisher Scientific, Waltham, MA, USA). Dissection medium for roGFP2 experiments was supplemented with 2 mM *N*-ethylmaleimide (NEM) to prevent additional oxidation. Brains were fixed in 3.7% formaldehyde (ThermoFisher Scientific), washed with 0.3% Triton X-100 in phosphate-buffered saline (PBT), and blocked in 10% goat serum (Invitrogen, Carlsbad, CA, USA). Brains from roGFP2-expressing flies were incubated overnight with 1.0% anti-TH antibodies purified from rabbit serum (#AB152MI, MilliporeSigma, Burlington, MA, USA) at 4°C. For nitration studies, brains were incubated in 1.0% nitro-tyrosine antibodies purified from rabbit serum at 4°C overnight (#A21285, ThermoFisher Scientific). The following day brains incubated in 0.5% Alexa Fluor 594 goat anti-rabbit IgG secondary antibodies (#A11012, ThermoFisher Scientific). Washed brains were mounted onto microscope slides using Invitrogen™ ProLong™ Diamond Antifade Mountant (ThermoFisher Scientific) mounting medium and cured overnight at ambient temperature. A detailed description of this protocol can be found in Buhlman et al. (2021).

### Image capture and analysis

For roGFP2 measurements, *z-*stacks of one PPL1 region per brain were captured at 630×magnification with a Stellaris confocal microscope (Lecia Microsystems, Wetzlar, Germany) and analyzed with Image Pro Premier 3D image processing software (Media Cybernetics, Inc., Rockville, MD, USA). Total volumes of fluorescence emission from oxidized (excited with 405 nm laser) and non-oxidized roGFP2 (excited with 488 nm laser) within the TH-labeled volume were calculated. Oxidized and non-oxidized roGFP2 emission ranges were 500-530 nm. Image Pro Premier 3D software generates ‘isosurfaces’ for fluorescence emission volumes above background. Image capture parameters and fluorescence thresholds for volume measurements were consistent for all samples, and the experimenter was unaware of the conditions during image analyses. To determine relative levels of hydrogen peroxide and G-SH redox equilibrium, the sum of the total volume of oxidized reporter per region was divided by emission volume for the non-oxidized reporter. For NOS-driven GFP and nitration antibody volume measurements, *z*-stacks of the central brain were captured at 200×, and the sums of anti-EGFP and secondary antibody emission volumes within the central brain were calculated as previously described (Jones et al., 2024). Differences in brain size were controlled for by dividing the volume of fluorophore emission by the volume of the central brain based on background secondary antibody staining. Sample size was calculated using preliminary data means and standard deviations assuming 0.05 for α and 0.8 for desired power. We used the ROUT method with a false discovery rate of 1% to remove outliers (GraphPad Prims 10) (Motulsky and Brown, 2006). Standard deviations were not different from one another for the hydrogen peroxide study (Brown−Forsythe test, *P*=0.1618). G-SH equilibrium data had unequal variances; therefore, the Brown−Forsythe test was repeated on square root-transformed data (*P*=0.1864; graph shows raw data and *P* values from analyses performed on transformed data). Nitration data also had unequal variance and, therefore, underwent logarithm transformation (Brown−Forsythe test, *P*=0.5844; graph shows original data and *P* values from analyses performed on transformed data). Brain size and dNOS data had equal variance (Brown−Forsythe test, *P*=0.0586 for brain size; *P*=0.1820). Two-way ANOVA followed by Tukey's multiple comparisons tests were performed to determine the effects of genotype and sex (GraphPad Prism 10). Each data point indicates the ratio for one PPL1 region per brain or one central brain. Means and standard errors are also plotted (*n*≥23).

Liquid chromatography tandem mass spectrometry (LC-MS/MS) analysis of low-molecular-weight thiols/disulfides

### Preparation of *Drosophila* brain homogenates for liquid chromatography tandem mass spectrometry (LC-MS/MS) analysis

Groups of 7-25 newly eclosed *park*^*+/+*^ or *park^−/−^* flies were transferred to fresh standard food and maintained at 25°C in 12 h of light with constant humidity. Heads were pooled by sex and genotype, and collected in 20 mM NEM in 15% methanol between days 4 and 6 post-eclosion. Heads were flash-frozen in liquid nitrogen and stored at −80°C.

### Chemicals and reagents for LC-MS/MS analysis

Low-molecular-weight reduced thiols [i.e. L-glutathione (G-SH), L-cysteine (Cys), L-homocysteine, L-methionine, and L-cystathionine], oxidized thiols [i.e. L-glutathione disulfide (GSSG) and L-cystine (CySS)] and NEM were received from Millipore Sigma. L-γ-glutamyl-L-cysteine and L-methionine-d3 were acquired from Cayman Chemical. L-cysteinylglycine, DL-cystine-d6, glutathione (glycine- 13C2, 15N) sodium salt, glutathione disulfide- 13C4, 15N2 ammonium salt, D,L-cystathionine-d4 and L-cysteine-13C3,15N were acquired from Toronto Research Chemicals and DL homocysteine-d4 was purchased from CDN Isotopes. Formic acid (>98%), HPLC or LC-MS-grade reagents and solvents [ammonium formate, methanol and acetonitrile (ACN)] were obtained from Fisher Scientific. The Milli-Q IQ 7000 filter system served as a source for LC-MS-grade H_2_O.

### Sample preparation for LC-MS/MS analysis

Fly heads were homogenized in a solution containing 20 mM NEM solution in 15% methanol in H_2_O and incubated at room temperature for 45 min to allow complete derivatization of thiols. A 30 µl aliquot of the sample or calibration curve standard solution was mixed with 130 µl ACN containing 0.1% formic acid (v/v) and internal standards. After centrifugation (12,000 ***g*** for 10 min), 5 µl of the supernatant was injected into the LC-MS system for analysis. For quantification of thiols and methionine, supernatants of respective samples were further diluted ten-fold prior to injection.

### LC-MS/MS analysis

Analytes were separated using an Intrada Amino Acid column (50 mm×2 mm, 3 μm; Imtakt Corporation Kyoto, Japan) on a SCIEX Exion LC UHPLC system (Foster City, CA, USA) operating in gradient elution mode. The mobile phases consisted of Phase A (10 mM ammonium formate in a 4:1 aqueous solution of H_2_O and ACN) and Phase B (0.1% formic acid in ACN) with a flow rate of 0.5 ml/min. Detection was performed on a Sciex QTRAP 6500+ mass spectrometer (Foster City) equipped with an electrospray ionization source, operating in multiple reaction monitoring (MRM) mode. Stable isotope-labeled internal standards were used for each analyte to ensure accurate quantification (Houlihan et al., 2022; Nagendla et al., 2022).

### Protein assay

Protein concentrations in samples were measured by using the Pierce Coomassie Plus (Bradford) Protein Assay Kit (Pierce Biotechnology, Rockford, IL, USA) by following manufacturer's instructions. Absorbance was recorded at 595 nm using a microplate reader (SPARK, Tecan Group Ltd). Thiol and disulfide concentrations were normalized to the corresponding protein concentrations.

### Statistical analyses

LMW thiol concentrations for pooled samples within the same experiment were normalized to those for control females. Sample size was calculated using preliminary data means and standard deviations assuming 0.05 for α and 0.8 for desired power. We used the ROUT method with a false discovery rate of 1% to remove outliers (GraphPad Prims 10) (Motulsky and Brown, 2006). With the exceptions of cysteine/cystine and cystine, LMW thiol data had equal variances; therefore, cysteine/cystine and cystine data underwent square root transformation to achieve equal variances (Brown−Forsythe test: *P*=0.2625 for cysteine/cystine, *P*=0.1581 for cystine). Two-way ANOVA followed by Tukey's multiple comparisons tests were performed to determine effects of genotype and sex (*n*≥8; GraphPad Prism 10). Untransformed data appear on graphs.
